# Supplementary material for: Exploring the Sensory Properties and Preferences of Fruit Wines Based on an Online Survey and Partial Projective Mapping
Source: Foods. 2023 Apr 29;12(9):1844. doi: 10.3390/foods12091844 (PMC10178241; doi:10.3390/foods12091844)

Supplementary Table S1 The types of fruit wines and their alcoholic content

	Blueberry wines				Apricot wine	Hawthorn wines			Rose roxburgh wine	Goji berry wine
Samples	B1	B2	B3	B4	A	H1	H2	H3	R	G
Alcohol Content （%）	12	12	12	10.5	17-19	15	8	12	12	7
Type	Dry	Sweet	Dry	Dry	Sweet	Dry	Sweet	Semi-sweet	Sweet	Semi-dry

Supplementary Table S2 Influence of gender on liking, familiarity, and usage ratings for 6 wines

		Gender		
		Liking (1-5)	Familiarity (1-5)	Consumption Frequency (1-5)
Grape wine	**1**	3.837±0.981a	3.535±1.014	2.395±0.786a
	**2**	3.520±0.795b	3.554±1.005	2.122±0.582b
Blueberry wine	**1**	3.558±0.889	2.581±1.057	1.558±0.606
	**2**	3.649±0.790	2.784±0.959	1.622±0.599
Hawthorn wine	**1**	3.244±1.005	2.384±0.923b	1.337±0.467b
	**2**	3.142±0.940	2.670±1.109a	1.520±0.633a
Goji berry wine	**1**	3.047±1.062	2.372±1.041	1.384±0.617
	**2**	2.878±0.880	2.622±1.072	1.405±0581
Rosa roxburghi wine	**1**	3.023±0.945	1.965±0.744	1.221±0.417
	**2**	3.020±0.782	2.034±0.844	1.203±0.801
Apricot wine	**1**	3.140±0.922	2.035±0.846	1.267±0.445
	**2**	3.142±0.800	2.108±0.842	1.277±0.590

Gender 1 is females (*n*=148) and 2 is males (*n*=86). Letters a-b show significant differences between subgroups within sample (ANOVA with Tukey's for genders; p

< 0.05).

Supplementary Figure S1. Questionnaire data analysis(n=234). Frequency distribution of the acceptable alcohol content(A), considerations when purchasing alcohol products(B), analysis of general health interest (C), analysis of food neophobia scale (D), analysis of attitudes for alcoholic drinks (E) and analysis of attitudes for sweetness (F).

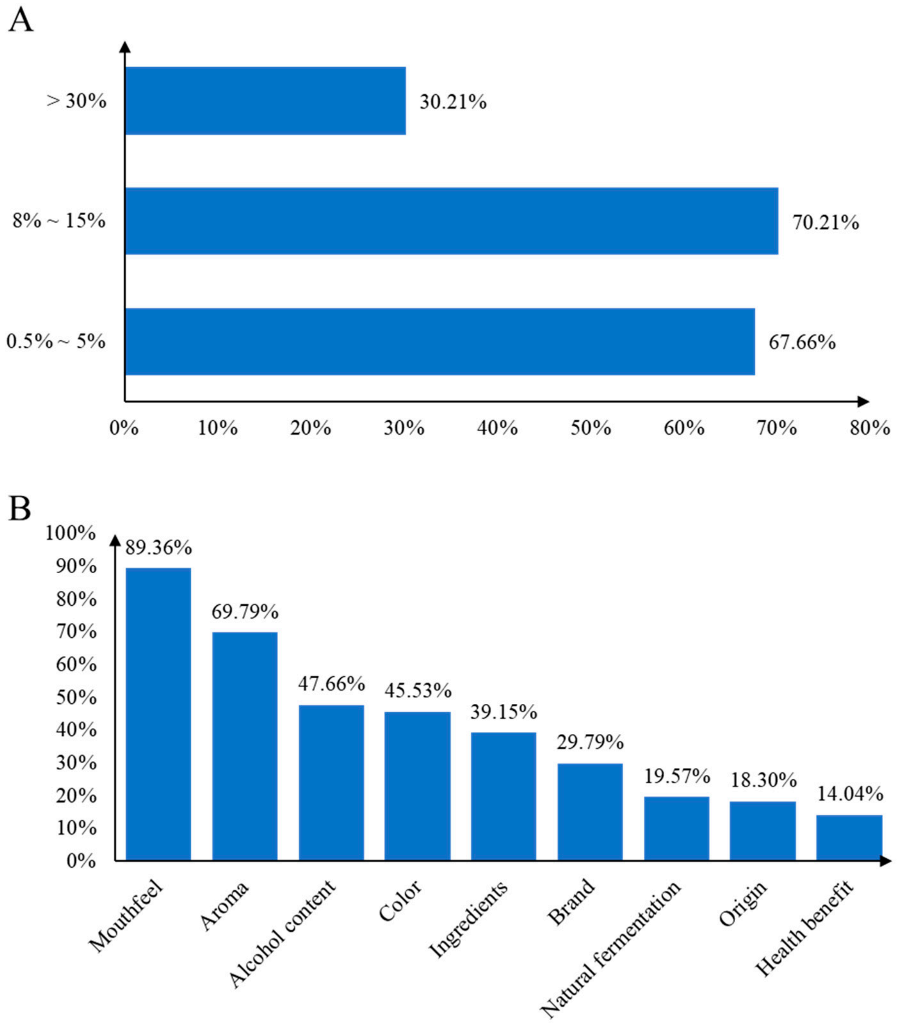

C

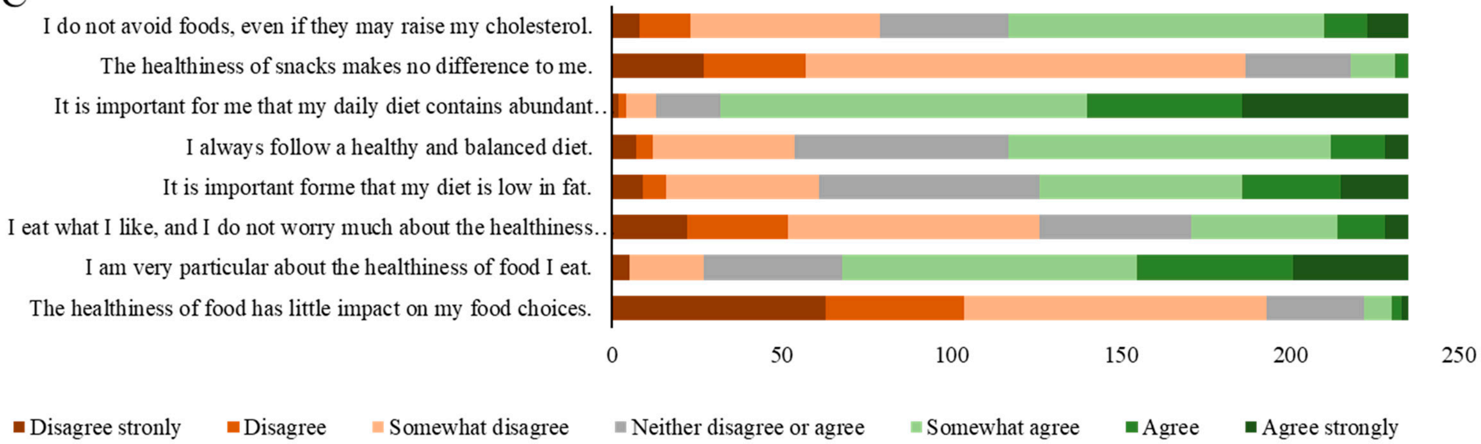
I do not avoid foods, even if they may raise my cholesterol.
The healthiness of snacks makes no difference to me.
It is important for me that my daily diet contains abundant..
I always follow a healthy and balanced diet.
It is important forme that my diet is low in fat.
I eat what I like, and I do not worry much about the healthiness..
I am very particular about the healthiness of food I eat.
The healthiness of food has little impact on my food choices.
0
50
100
150
200
250
Disagree stronly
Disagree
Somewhat disagree
Neither disagree or agree
Somewhat agree
Agree
Agree strongly

D

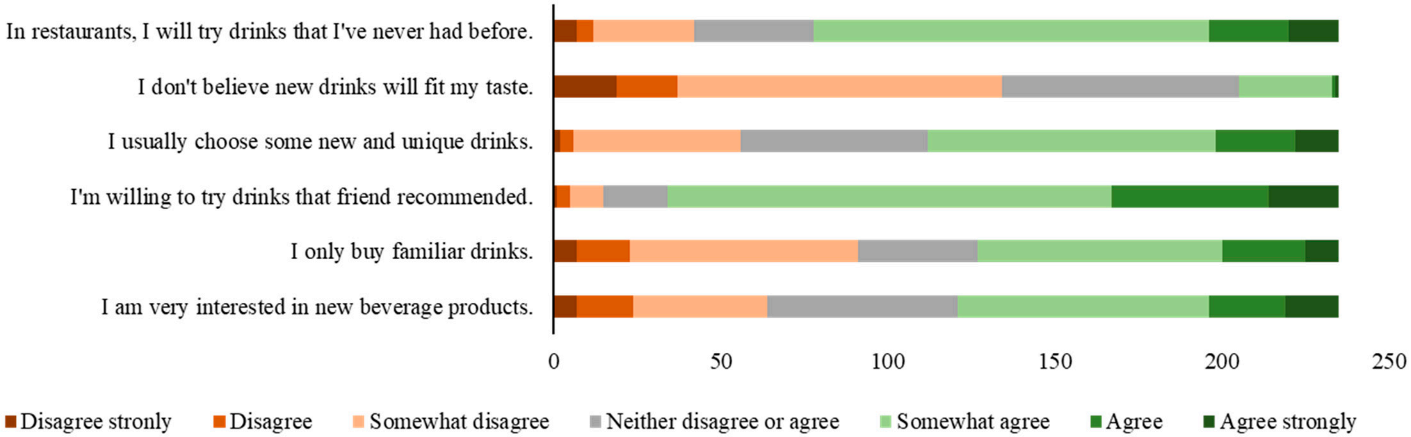
In restaurants, I will try drinks that I've never had before.
I don't believe new drinks will fit my taste.
I usually choose some new and unique drinks.
I'm willing to try drinks that friend recommended.
I only buy familiar drinks.
I am very interested in new beverage products.
0
50
100
150
200
250
Disagree stronly
Disagree
Somewhat disagree
Neither disagree or agree
Somewhat agree
Agree
Agree strongly

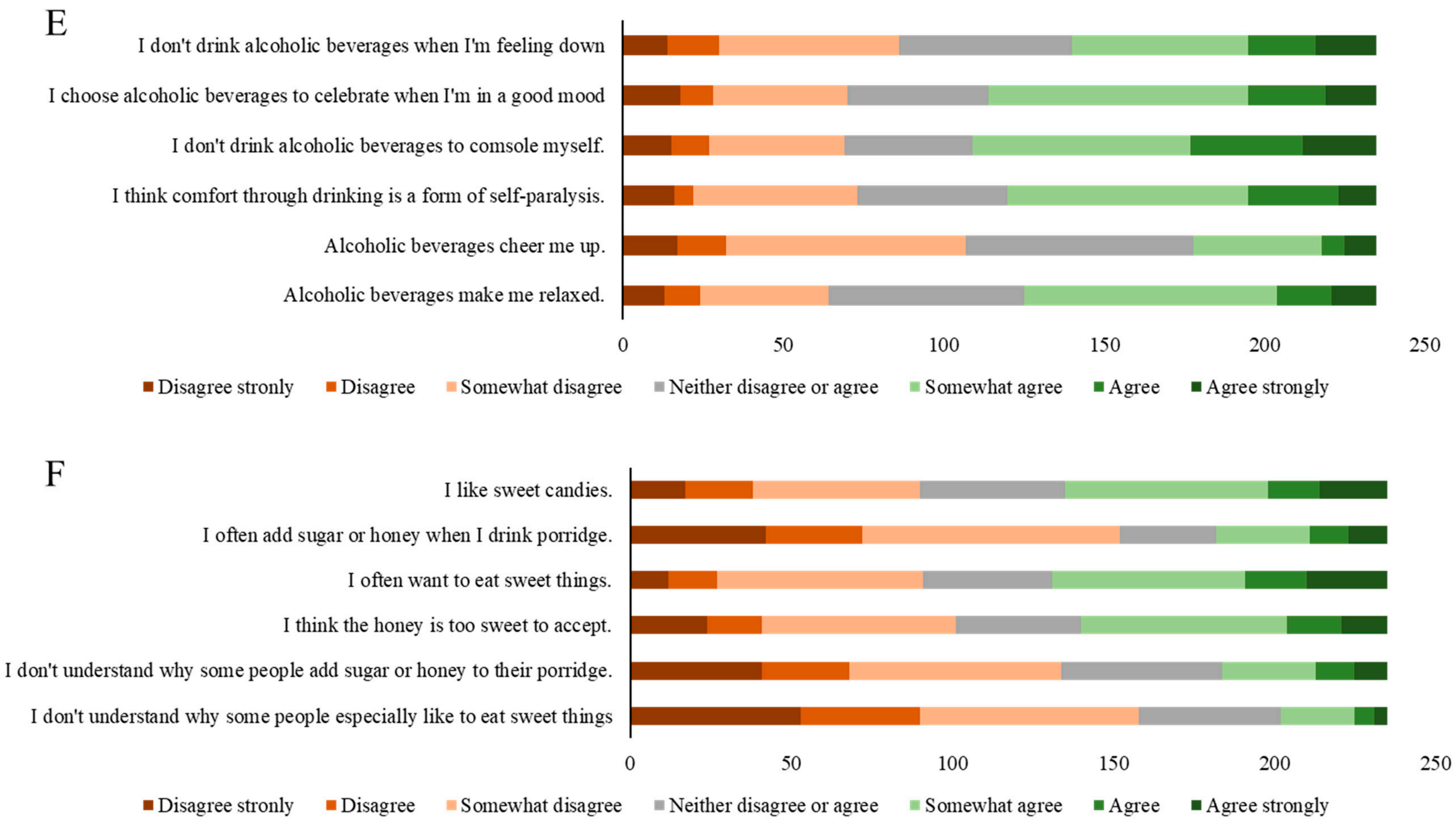

Supplementary Figure S2. Projective Mapping task word cloud with (A) appearance, (B) aroma and (C)taste.

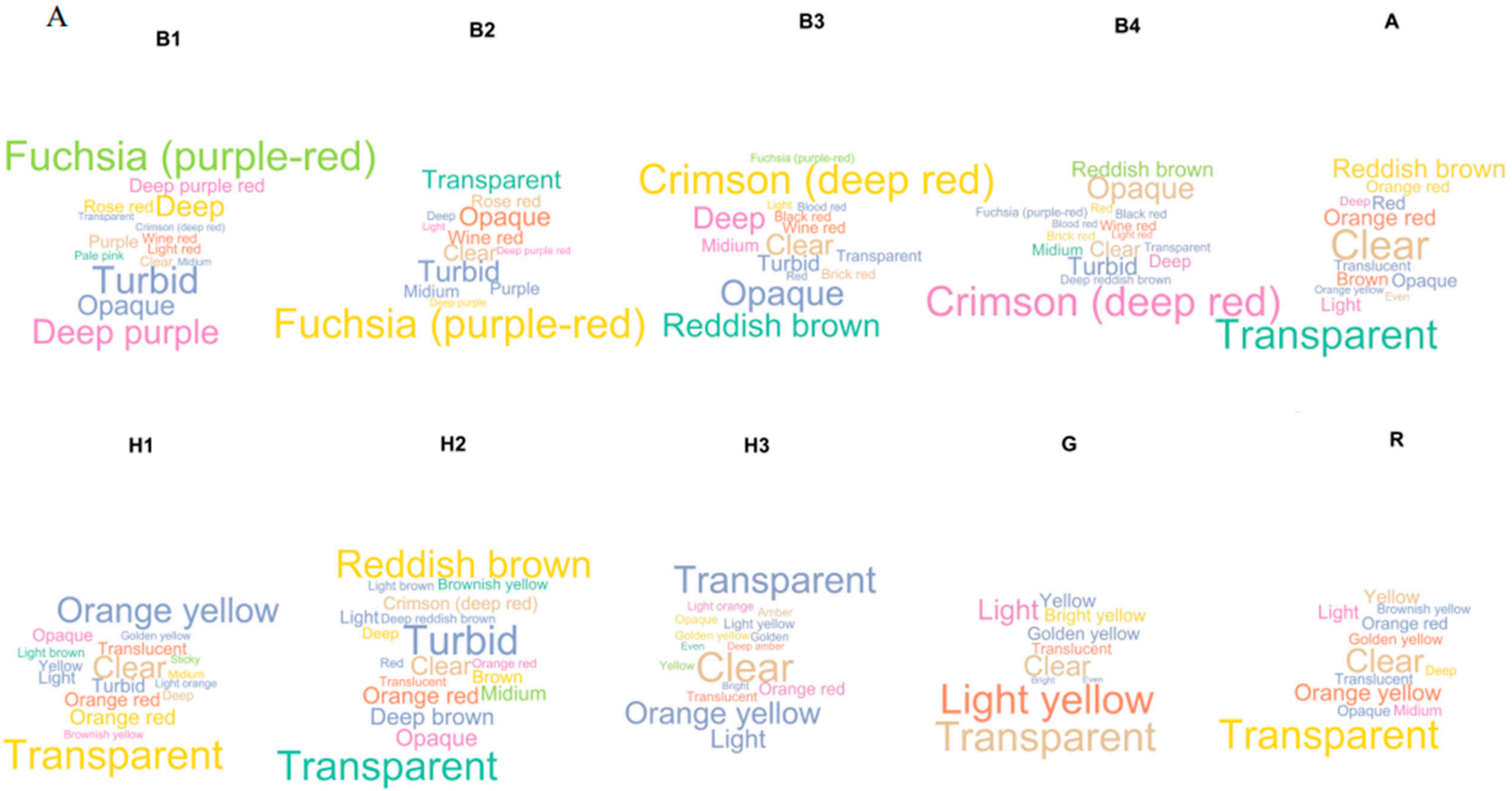
A
B1
Fuchsia (purple-red)
Deep purple red
Rose red
Deep
Purple
Wine red
Light red
Pale pink
Turbid
Opaque
Deep purple
B2
Transparent
Rose red
Opaque
Wine red
Clear
Turbid
Midium
Purple
Fuchsia (purple-red)
B3
Crimson (deep red)
Deep
Wine red
Midium
Clear
Turbid
Transparent
Opaque
Reddish brown
B4
Reddish brown
Opaque
Fuchsia (purple-red)
Black red
Midium
Clear
Transparent
Turbid
Deep
Deep reddish brown
Crimson (deep red)
A
Reddish brown
Orange red
Red
Orange red
Clear
Translucent
Brown
Opaque
Light
Transparent
H1
Orange yellow
Opaque
Golden yellow
Light brown
Translucent
Yellow
Clear
Light
Turbid
Orange red
Orange red
Transparent
H2
Reddish brown
Light brown
Brownish yellow
Crimson (deep red)
Light
Deep reddish brown
Deep
Turbid
Red
Clear
Orange red
Translucent
Brown
Orange red
Midium
Deep brown
Opaque
Transparent
H3
Transparent
Light orange
Light yellow
Golden
Clear
Translucent
Orange red
Orange yellow
Light
G
Light
Yellow
Bright yellow
Golden yellow
Translucent
Clear
Light yellow
Transparent
R
Yellow
Light
Brownish yellow
Orange red
Golden yellow
Clear
Deep
Translucent
Orange yellow
Opaque
Midium
Transparent

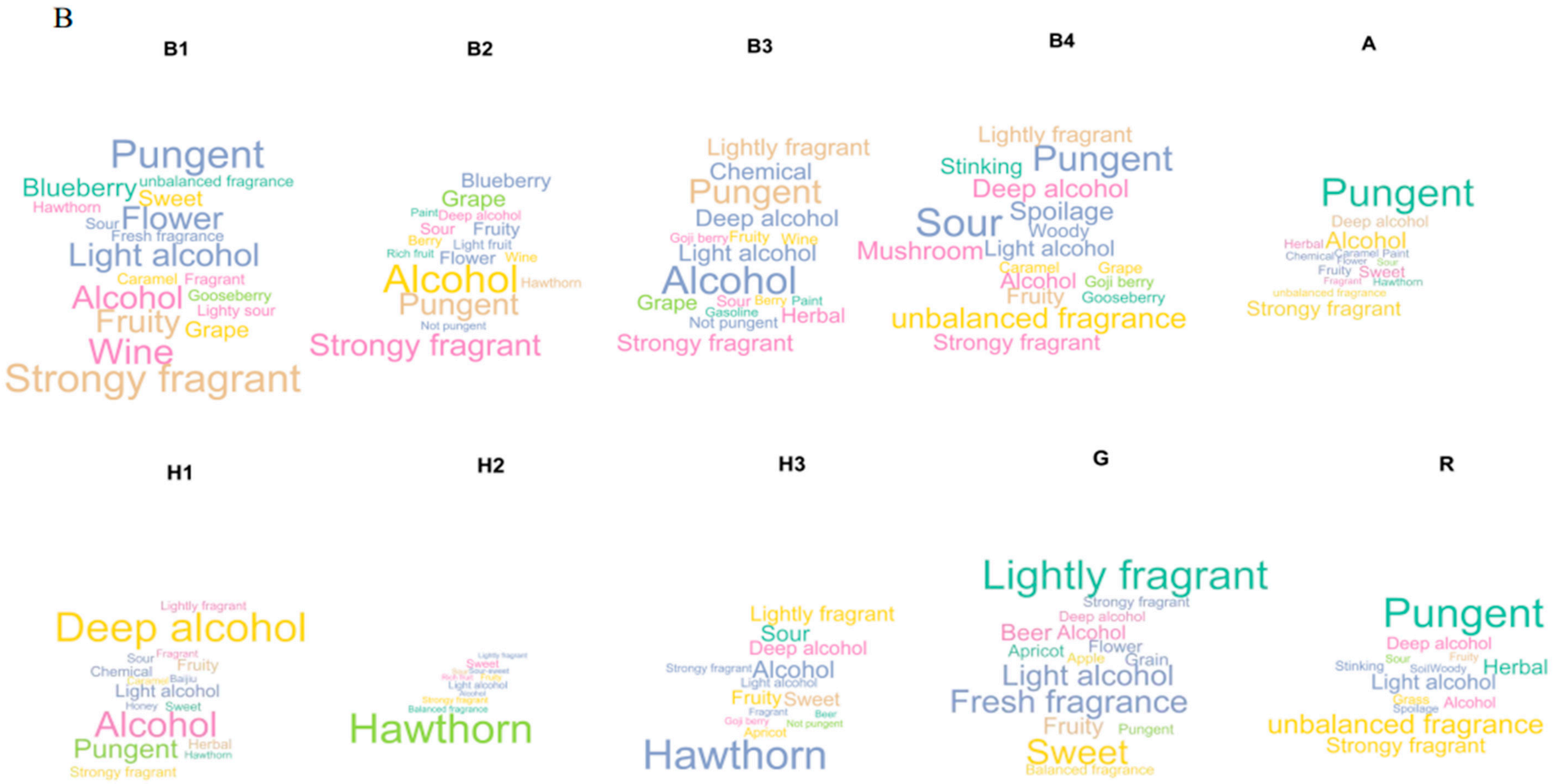
B
B1
Pungent
Blueberry
unbalanced fragrance
Sweet
Hawthorn
Sour
Flower
Fresh fragrance
Light alcohol
Caramel
Fragrant
Alcohol
Gooseberry
Lighty sour
Fruity
Grape
Wine
Strongy fragrant
B2
Blueberry
Grape
Paint
Deep alcohol
Sour
Fruity
Berry
Light fruit
Rich fruit
Flower
Wine
Alcohol
Hawthorn
Pungent
Not pungent
Strongy fragrant
B3
Lightly fragrant
Chemical
Pungent
Deep alcohol
Goji berry
Fruity
Wine
Light alcohol
Alcohol
Grape
Sour
Berry
Paint
Gasoline
Not pungent
Herbal
Strongy fragrant
B4
Lightly fragrant
Stinking
Pungent
Deep alcohol
Sour
Spoilage
Woody
Mushroom
Light alcohol
Caramel
Grape
Alcohol
Goji berry
Fruity
Gooseberry
unbalanced fragrance
Strongy fragrant
A
Pungent
Deep alcohol
Herbal
Alcohol
Chemical
Caramel
Paint
Flower
Sour
Fruity
Sweet
Fragrant
Hawthorn
unbalanced fragrance
Strongy fragrant
H1
Lightly fragrant
Deep alcohol
Sour
Fragrant
Chemical
Fruity
Caramel
Baijiu
Light alcohol
Honey
Sweet
Alcohol
Herbal
Pungent
Hawthorn
Strongy fragrant
H2
Sweet
Light alcohol
Balanced fragrance
Hawthorn
H3
Lightly fragrant
Sour
Deep alcohol
Strongy fragrant
Alcohol
Light alcohol
Fruity
Sweet
Fragrant
Beer
Goji berry
Not pungent
Apricot
Hawthorn
G
Lightly fragrant
Strongy fragrant
Deep alcohol
Beer
Alcohol
Apricot
Flower
Apple
Grain
Light alcohol
Fresh fragrance
Fruity
Pungent
Sweet
Balanced fragrance
R
Pungent
Deep alcohol
Stinking
Sour
Fruity
Soil
Woody
Herbal
Light alcohol
Grass
Spoilage
Alcohol
unbalanced fragrance
Strongy fragrant

C

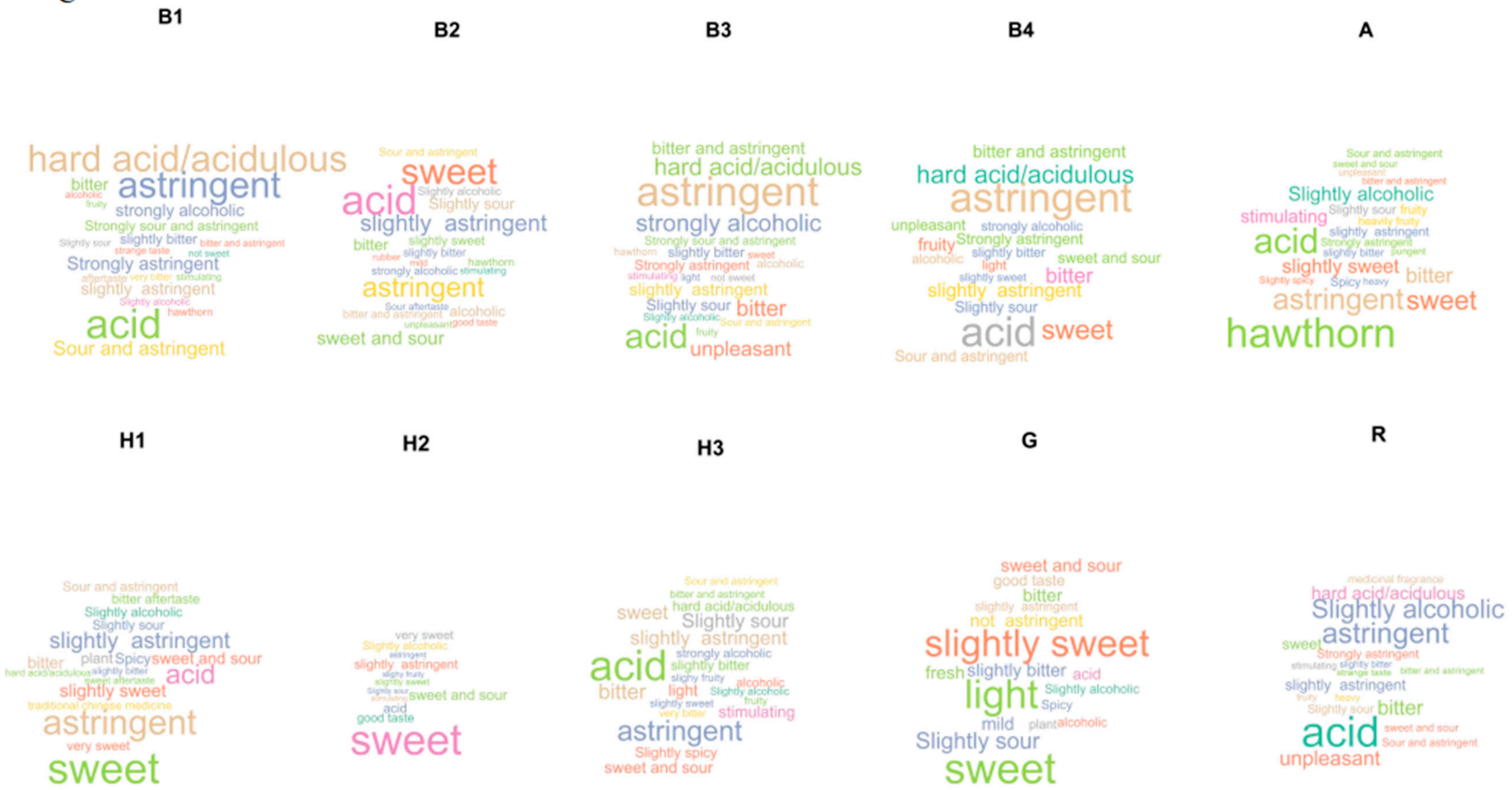
B1
hard acid/acidulous
astringent
bitter
strongly alcoholic
Strongly sour and astringent
slightly bitter
Strongly astringent
slightly astringent
acid
Sour and astringent
B2
sweet
acid
Slightly sour
slightly astringent
bitter
slightly sweet
astringent
alcoholic
sweet and sour
B3
bitter and astringent
hard acid/acidulous
astringent
strongly alcoholic
slightly astringent
Slightly sour
bitter
acid
unpleasant
B4
bitter and astringent
hard acid/acidulous
astringent
unpleasant
fruity
Strongly astringent
sweet and sour
bitter
slightly astringent
Slightly sour
acid
sweet
Sour and astringent
A
Slightly alcoholic
stimulating
acid
slightly sweet
bitter
astringent
sweet
hawthorn
H1
Sour and astringent
bitter aftertaste
Slightly alcoholic
Slightly sour
slightly astringent
bitter
Spicy
sweet and sour
acid
slightly sweet
astringent
sweet
H2
very sweet
slightly astringent
sweet and sour
good taste
sweet
H3
sweet
hard acid/acidulous
Slightly sour
slightly astringent
acid
bitter
light
stimulating
astringent
Slightly spicy
sweet and sour
G
sweet and sour
good taste
bitter
not astringent
slightly sweet
fresh
Slightly bitter
acid
light
Slightly alcoholic
mild
Slightly sour
sweet
R
hard acid/acidulous
Slightly alcoholic
astringent
sweet
slightly astringent
bitter
acid
unpleasant

Supplementary Figure S3. Liking: no consumer segmentation-2 clusters.

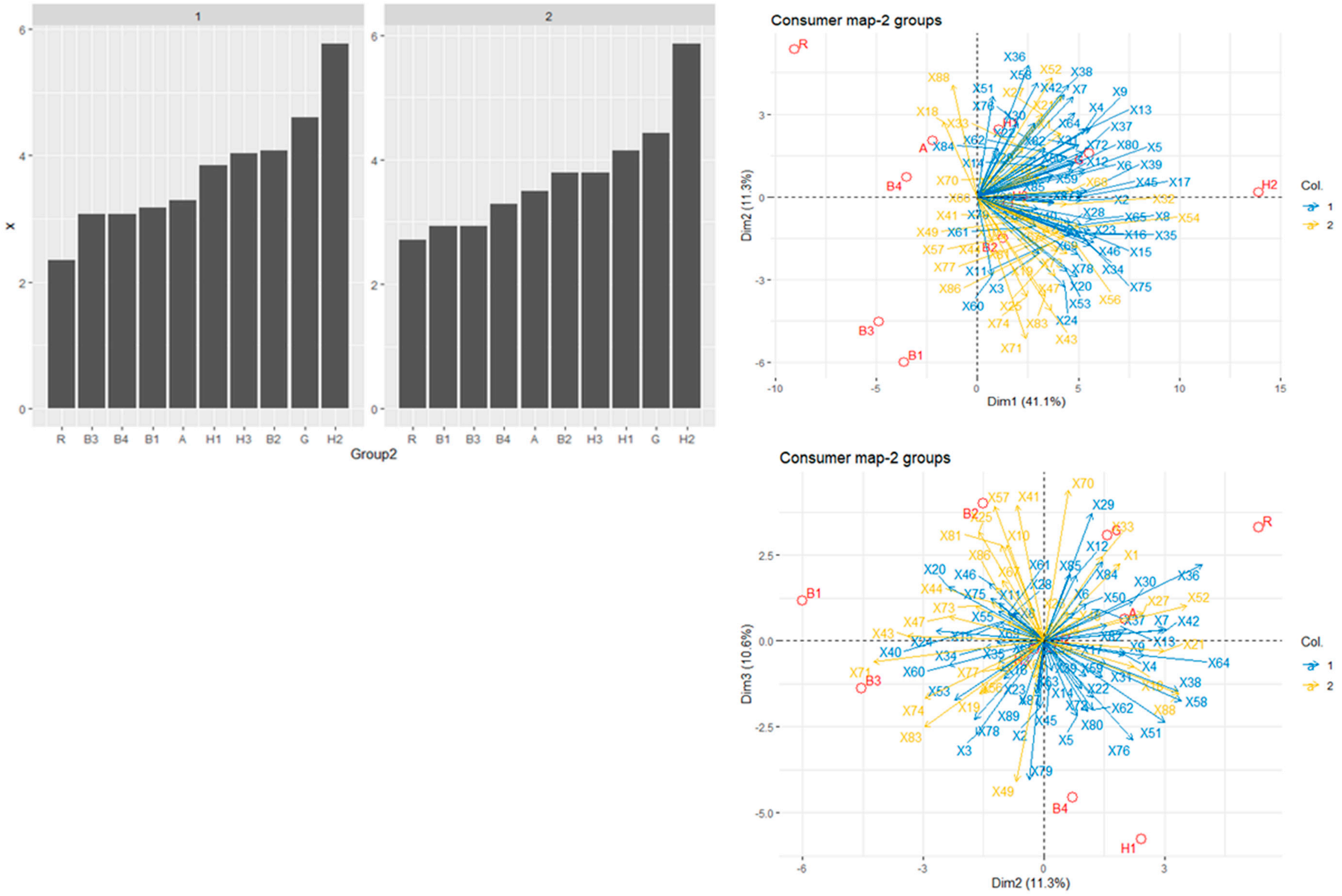

Supplement: Supplementary file 1 [file foods-12-01844-s001.zip › foods-2270572-supplementary.pdf]
